# Experimental Study on the Shear Strength and Failure Mechanism of Cemented Soil–Concrete Interface

**DOI:** 10.3390/ma16124222

**Published:** 2023-06-07

**Authors:** Jie Zhou, Chao Ban, Huade Zhou, Junjie Ren, Zhong Liu

**Affiliations:** 1 Department of Geotechnical Engineering, College of Civil Engineering, Tongji University, 1239 Siping Road, Shanghai 200092, China; 2230201@tongji.edu.cn (C.B.); 2210401@tongji.edu.cn (H.Z.); 1930175@tongji.edu.cn (J.R.); 2 Key Laboratory of Geotechnical and Underground Engineering, Ministry of Education, Tongji University, 1239 Siping Road, Shanghai 200092, China; 3 Zhejiang Kunde Innovate Geotechnical Engineering Co., Ltd., Ningbo 315000, China; liuz@kundeyt.com

**Keywords:** cemented soil–concrete interface, large-scale interface shear test, interface shear strength, unconfined compressive strength

## Abstract

Cement is always used in underground construction to reinforce and improve soft clay, resulting in the formation of a cemented soil–concrete interface. It is of great importance to study interface shear strength and failure mechanisms. So, in order to figure out the failure mechanism and characteristics of a cemented soil–concrete interface, a series of large-scale shear tests of a cemented soil–concrete interface, and corresponding unconfined compressive tests and direct shear tests of cemented soil, were carried out specifically under different impact factors. A kind of bounding strength was observed during large-scale interface shearing. Resultantly, three stages of the shear failure process of the cemented soil–concrete interface are proposed, and bonding strength, peak (shear) strength and residual strength are pointed out, respectively, in interface shear stress–strain development. Based on the analysis results of the impact factors, the shear strength of the cemented soil–concrete interface increases with age, the cement mixing ratio and normal stress, and decreases with the water–cement ratio. Additionally, the interface shear strength grows much more rapidly after 14 d to 28 d compared to the early stage (1~7 d). Additionally, the shear strength of the cemented soil–concrete interface is positively related to unconfined compressive strength and shear strength. However, the trends of the bonding strength and unconfined compressive strength or shear strength are much closer than those of the peak and residual strength. This is considered to be related to the cementation of cement hydration products and probably the particle arrangement of the interface. Particularly, the cemented soil–concrete interface shear strength is always smaller than the cemented soil’s own shear strength at any age.

## 1. Introduction

Interfaces are important in all kinds of geotechnical and underground engineering structures [1,2,3,4]. Since the mechanical properties of two materials in contact with each other are different, and the interface is also the main carrier for the interaction between them, large shear stress and differential deformation are easily generated at the interface. In such cases, this will most likely lead to structural failure and instability. Therefore, the study of the shear characteristics of interfaces has always been an important research topic in geotechnical and underground engineering. 

In underground projects, an interface between the soil and structures (soil–concrete interface) is always formed. Additionally, many studies have been conducted on soil–concrete interfaces. Gong [5] studied the effect of normal-stress history on the shear characteristics of a clay–concrete interface by using a large direct shear system, and found that the shear stress would be larger at the same shear displacement with increasing initial normal stress. Additionally, the larger the initial normal stress was, the larger the maximum shear stress at the interface would be. Liu [6] conducted an experimental study on the shear properties of a silty clay–concrete interface under freeze–thaw cycles, and found that the shear strength of the interface was positively related to normal stress and negatively related to the number of freeze–thaw cycles and the initial water content of the soil. Namdar [7] found that the differential settlement of soil depends on the soil–concrete foundation interaction through a numerical investigation on soil–concrete foundation interactions.

**Figure 1:**
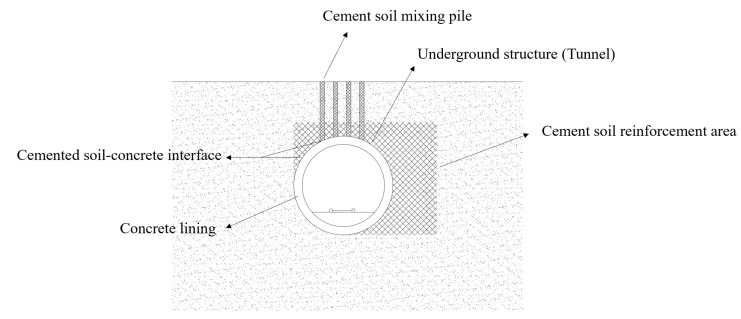
Diagram of the cemented soil–concrete interface of underground structures.

However, in coastal areas such as Shanghai, Tianjin and Ningbo, soft clay is widely distributed. Their engineering properties of low strength, high compressibility and low permeability substantially increase the difficulty of engineering construction. In order to effectively improve the properties of soft clay, cement is usually used in practical construction to reinforce and improve the soil, such as cement mixing pile [8,9], high-pressure rotary jet grouting pile, concrete-cored DCM pile [10], etc. Even in the process of the artificial ground freezing method (AGF), in order to reduce the impact of frost heave and thaw collapse, the soil will also be grouted to improve it. In these projects, there will be a cemented soil–concrete interface formed between the cemented soil and some underground structures, as shown in Figure 1. 

The cemented soil–concrete interface plays a significant role in the structure’s overall structural stability. However, the cemented soil is often cast in situ. Its strength is generally influenced by its age, the cement mixing ratio, the water–cement ratio, etc., which will definitely further influence the performance of the shear strength of the cemented soil–concrete interface. Zhou [11] studied the behavior of pre-bored grouting planted piles under compression and tension, and found that the frictional capacity of the concrete–cemented soil interface was mainly controlled by the properties of the cemented soil. Correia [12] and Horpibulsuk [13], respectively, studied chemically stabilized soft soils and cement-admixed high-water-content clays. It was found that the strength of cemented soil was related to the binder content, the liquidity index and clay-water/cement ratio. Considering the existence of concrete–cemented soil interfaces in underground constructions, there is no doubt that the study of the characteristics of these interfaces cannot be neglected because of the bond forces generated by cement. Tanchaisawat [14] studied the characteristics of cemented soil–concrete interfaces through shear testing. The results showed that the interface shear strength increased linearly with the unconfined compressive strength of the cemented soil. Wu [15] conducted laboratory tests on the interface of cemented soil–concrete and found that the value of interface shear strength was about 0.194 times the unconfined compressive strength of the cemented soil specimen. Peng [16] concluded that the interfacial shear strength of cement-treated soil and concrete was about 0.188 times the unconfined compressive strength of cement-treated soil. Jamsawang [17] conducted a pullout test of a concrete core pile in the field of a stiff composite pile, and the results showed that the interfacial shear strength could be 0.4 times the unconfined compressive strength of the cement pile. Li [18] took the effect of normal stress into consideration, and established an empirical equation between the shear strength of a cemented soil–concrete interface and normal stress. Yu [19] conducted an experimental study on the frictional capacity of the concrete–cemented soil interface of a concrete-cored cemented soil column and found that the relationship between the ultimate lateral friction and the unconfined compressive strength of the cemented soil was approximately linear. Zhou [20] analyzed the interface shear characteristics of concrete pile body and cemented soil by examining the pile axial force and load–settlement curve in a model test of pre-bored pile. Additionally, it was concluded that the concrete-cemented soil interface strength was much greater than the cemented soil–soil interface strength. The authors of [21] then conducted an experimental study on the strength characteristics of a concrete–cemented soil interface. The experimental results showed that the interface strength had a positive correlation with the strength of cemented soil. 

However, the above studies mainly concentrate on analyzing the relationship between the shear strength of the cemented soil–concrete interface and the unconfined compressive strength of the cemented soil. The failure mechanism and shear characteristics of cemented soil–concrete interfaces are not clarified. At the same time, in some codes and specifications, such as the “Technical specification for strength composite piles” (DGJ32/TJ 151-2013) [22], the bearing capacity of the pile is determined by the frictional force at the cemented soil–concrete and cemented soil–soil interface. However, the friction at the interface is always determined by local experience. The influence of the real interface characteristics on the bearing capacity of the pile is not reflected in such a specification. Additionally, the mechanism of interaction between cemented soil and underground structures is not clear. Therefore, this paper discusses the effects of cemented soil strength and normal stress on the shear strength of a cemented soil–concrete interface through a series of large-scale shear tests. From the perspective of cemented soil strength, the influences of age, the cement mixing ratio and the water–cement ratio are also investigated. In order to establish a more practical method for estimating the shear strength of the cemented soil–concrete interface later, the shear strength of cement–soil is also considered, and a comprehensive analysis of the relationship between the shear strength of the cemented soil–concrete interface, the unconfined compressive strength and the shear strength of the cemented soil is carried out. The research results of this paper could provide references for future studies about the shear characteristics of cemented soil–concrete interfaces and a better understanding of the interface failure mechanism. Additionally, it will provide a basis for the design of underground construction involving cemented soil–concrete interfaces.

## 2. Materials and Methods

### 2.1. Materials

#### 2.1.1. Soil In Situ

**Figure 2:**
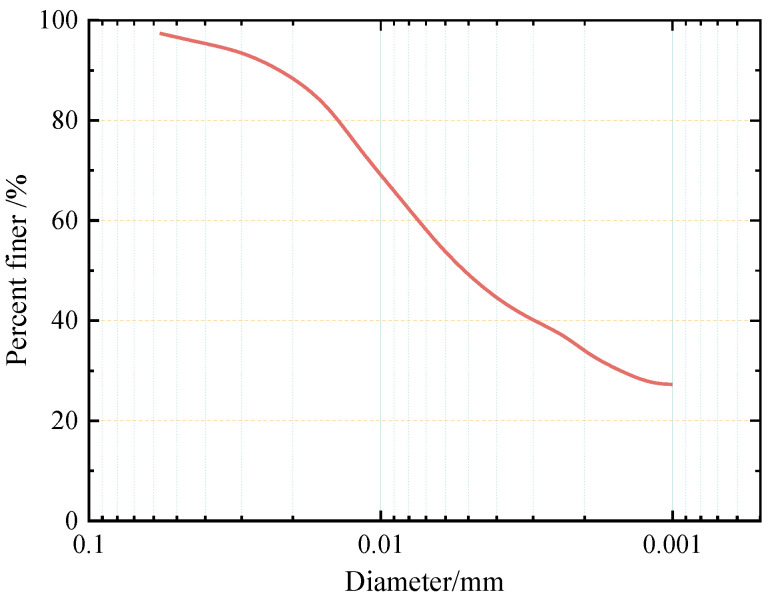
Gradation of soil sample.

The lengths of cement soil mixing piles and stiffened deep cement mixing piles used in engineering practice normally range from 10 m to 20 m [23,24]. Additionally, underground structures such as subway tunnels are also distributed from 10 m to 30 m underground. According to the geologic investigation data of Ningbo [25], a large amount of mucky soft clay is distributed along the piles. Therefore, the soil used in this research was mucky soft clay of the second layer in Ningbo, from an approximate depth of 12 m. The grain gradation curves of the soil samples are shown in Figure 2 and their basic physical properties are shown in Table 1.

#### 2.1.2. Cemented Soil 

Considering that the large-scale shear tests needed a large quantity of the cemented soil sample, the soil sample used in this paper was remolded soil. According to the “Specification for mix proportion design of cement soil” (JGJ/T 233-2011) [26], the air-dried soil sample was crushed and sieved. Then, the water content of air-dried soil sample was measured as $w_{0}$ and the mass ratio of cement to soil was measured as $a_{w}$. Additionally, $a_{a}$ represented the mass ratio of admixture to cement. The mass of air-dried soil, cement, water and admixture needed to configure cemented soil was calculated using Equations (1)–(4) while referring to the “Specification for mix proportion design of cement soil” (JGJ/T 233-2011) [26]. For Equation (1), according to the equal mass of soil particles in wet soil and air-dried soil, the mass of air-dried soil could be calculated. Additionally, the mass of cement and admixture could be obtained based on the definition of cement mixing ratio and admixture content in Equations (2) and (4). For Equation (3), the mass of water was calculated in two parts. The mass of water required in air-dried soil was calculated in the first part according to the difference in water content. At the same time, the required water in cement was calculated according to the definition of the water–cement ratio.

(1) $$m_{0}=\frac{1+0.01w_{0}}{1+0.01w}m_{s}$$

(2) $$m_{c}=\frac{1+0.01w}{1+0.01w_{0}}0.01a_{w}m_{0}$$

(3) $$m_{w}=\frac{0.01w-0.01w_{0}}{1+0.01w_{0}}m_{0}+0.01\mu a_{w}m_{s}$$

(4) $$m_{a}=0.01a_{a}m_{c}$$

where $w_{0}$ is the water content of air-dried soil, %; w is the natural water content, %; $m_{s}$ is the mass of wet soil, kg; $m_{0}$ is the mass of air-dried soil, kg; $a_{w}$ is the cement mixing ratio, %; $m_{c}$ is the mass of cement, kg; $m_{w}$ is the mass of water, kg; μ is the water–cement ratio; $a_{a}$ is the admixture content, %; and $m_{a}$ is the mass of admixture, kg. 

Additionally, the cement type used was P42.5 ordinary Portland cement, which is commonly used in construction. Its properties are shown in Table 2.

#### 2.1.3. Concrete Slab

C80 high-strength concrete is always used in underground engineering. According to “Specification for mix proportion design of ordinary concrete” (JGJ 55-2011) [27] and “Technical specification for high performance concrete” (GB/T 41054-2021) [28], concretes of different proportions were test-matched. Hence, the concrete samples were configured as presented in Table 3. In addition to the samples to be tested with sizes of 600 × 400 × 100 mm, three cubes with sizes of 70.7 × 70.7 × 70.7 mm were also prepared for unconfined compression tests. Through unconfined compression tests on concrete, the average compressive strength of cubes was found to be 85.7 MPa. Steel molds with sizes of 600 × 400 × 100 mm were used to cast the concrete samples.

### 2.2. Experimental Program and Sample Preparation

#### 2.2.1. Large-Scale Shear Tests of Cemented Soil–Concrete Interface

(1) Experimental apparatus and program

**Figure 3:**
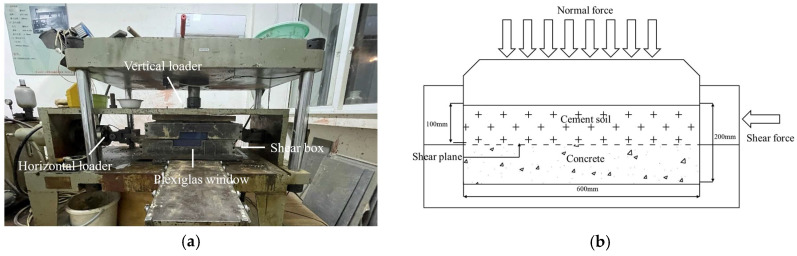
Large-scale interface shear apparatus. (**a**) Large-scale interface shear apparatus; (**b**) Schematic diagram of loading mechanism.

In order to decrease the size effect, large-scale shear tests were carried out to measure the shear strength of the cemented soil–concrete interface. According to the “Standard for test methods of engineering rock mass” (GB/T 50266-2013) [29], the test apparatus used was designed by the Department of Geotechnical Engineering College of Tongji University, and is shown in Figure 3. Firstly, the cemented soil–concrete sample was placed in a shear box with the concrete on the bottom and the cemented soil on the top. After the vertical load was applied, the strain control method was employed during the shear process at a rate of 1.0 mm/min. When the shear stress was almost stable, the shear test was completed. The performance parameters of this apparatus were a maximum normal load of 100 kN with precision of 0.1 N; the largest shear displacement of ±75 mm with precision of 0.1 mm; and a shear rate of 0.1~10 mm/min. 

Taking the soil in situ properties into account, normal stresses of 50 kPa, 150 kPa, and 250 kPa were considered in the experimental design. Considering the influencing factors of cemented soil strength, we set age, cement mixing ratio, water–cement ratio and normal stress as impact factors to discuss the interface shear characteristics. Since the increase in cement strength grows greatly in the early stage and becomes much more stable after 28 d, in this experiment, the age division was short initially and became longer afterwards. So, 1 d, 2 d, 3 d, 7 d, 14 d and 28 d were, respectively, set as the age factors. The cement mixing ratio and water–cement ratio were set based on both specifications, technical codes (shown in Table 4) and local engineering experience. In the Ningbo area, the common values of the cement mixing ratio and the water–cement ratio in engineering are 15% and 0.5, respectively. At the same time, in order to increase the fluidity of cemented soil, 0.01% polycarboxylate superplasticizer is also commonly added in engineering practices. Therefore, in this experiment, 15% was used as the reference value of the cement mixing ratio, and it fluctuated by one level smaller and larger (13%, 18%), respectively (based on the smallest 13% in the cement mixing pile in the specifications, as shown in Table 4), to analyze the influence of the cement mixing ratio on the cemented soil strength parameters. Additionally, the fluidity of the cemented soil was poor when the water–cement ratio was 0.5. If the water–cement ratio decreased, it would lead to a decrease in fluidity, making it difficult to ensure the uniformity of the cemented soil sample. Thus, combined with the recommended value range in Table 4, 0.5 was used as the reference value, and two levels (0.8, 1.0) were designed to analyze the influence of the water–cement ratio on cemented soil strength parameters. Considering that the preparation of large-scale cemented soil–concrete interface samples is time- and labor-consuming, and the water–cement ratios commonly used in engineering practice are between 0.5 and 0.8, in the large-scale interface shear tests, only two levels of 0.5 and 0.8 were set for the water–cement ratio factor. In order to investigate the effect of age, cement mixing ratio, water–cement ratio and normal stress on the shear strength of the cemented soil–concrete interface, a detailed experimental program of large-scale shear tests of the cemented soil–concrete interface was designed and is shown in Table 5, in which 28 d, 15%, 0.5 and 150 kPa are the basic reference parameter values.

(2) Preparation of cemented soil–concrete interface sample

**Figure 4:**
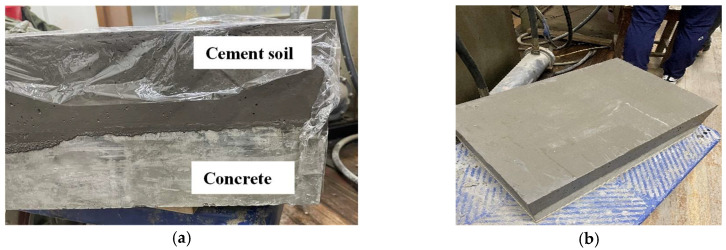
Cemented soil–concrete interface sample. (**a**) Interface sample before curing, after preparation; (**b**) Interface sample before testing, after curing.

The size of the shear box used in the large-scale shear test was 600 × 400 × 200 mm, and molds of same size were also prepared. Firstly, the concrete slab, which had reached curing age, was placed on the bottom of the mold, and we applied a thin layer of Vaseline around the mold. Then, the configured cemented soil was filled into the mold in layers and covered the concrete slab. Because the sample was relatively large, a vibrating rod was used to ensure the cemented soil and concrete were well compacted and avoid the formation of large bubbles inside. Finally, the surface of the cemented soil was scraped, and the sample was cured for 24 h before demolding. The entire set of cemented soil–concrete standard samples (Figure 4) were put in a curing room for a specified amount of time.

(3) Specimen quality control

**Figure 5:**
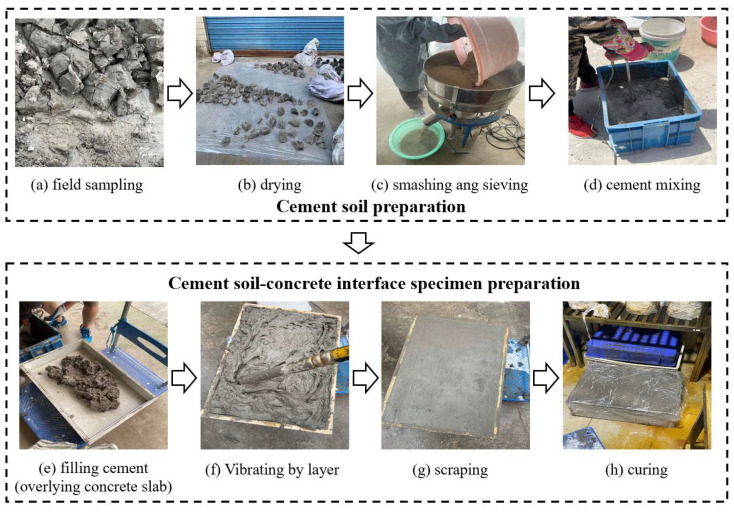
Large-scale cemented soil–concrete interface specimen preparation and quality control procedures.

It was necessary for the specimen quality to be strictly controlled throughout the experiments. Density control was conducted and best uniformity determined for sample preparation. Firstly, the soils were sampled in situ (Figure 5a) and totally dried in the air (Figure 5b); then, they were smashed and sieved into a uniform soil powder (Figure 5c). Most importantly, according to the water content and soil density of field soil (Table 1) and the specifically designed cement mixing ratio and water–cement ratio (Table 5), the total mass of air-dried soil, cement, water and admixture was calculated based on Equations (1)–(4) for the cemented soil–concrete interface sample volume. Additionally, we then mixed these materials together (Figure 5d). In this step, uniformity was very important, as well, for specimen quality control. A large high-speed motor stirrer was used for initial cement mixing. Additionally, then, the mass of each sublayer was determined and filled into a mold overlying a concrete slab (Figure 5e). Here, it was necessary for the density to be strictly controlled, i.e., a certain mass of cement should be weighed and totally filled into a predesigned sublayer volume. During this process, a shaking machine was also used to remove air bubbles in the cemented soil via overall shaking. In addition, a high-speed vibrating tube was used to help all the cement to be poured without any voids, vibrating it locally piece by piece, especially ambient walls. After all the layers were sub-filled, the specimen was scraped and subsequently maintained in a curing chamber for a specific amount of time, as shown in Table 5. Finally, small specimens were kept for strength comparison with the field samples during cement mixing pile construction. 

#### 2.2.2. Unconfined Compression Test and Direct Shear Test of Cemented Soil

Corresponding to the large-scale interface shear tests, unconfined compression tests and direct shear tests of the cemented soil were also designed and conducted under the same impact factors of age, cement content, water–cement ratio and normal stress, according to the “Standard for geotechnical testing method” (GB/T 50123-2019) [31]. The only difference was that the water–cement ratio was set at three levels of 0.5, 0.8 and 1.0 since the preparation was much easier, and the normal stress could be applied in stages. Therefore, the whole experimental program of the unconfined compression test and the direct shear test of cemented soil is shown in Table 6. 

**Figure 6:**
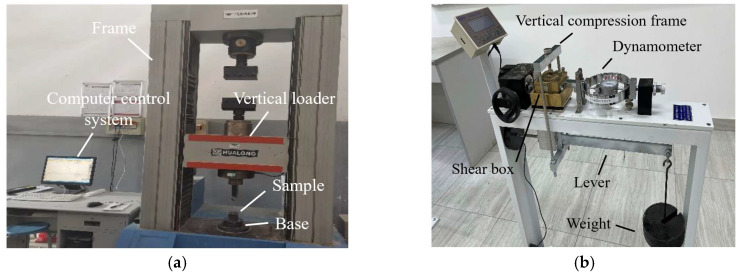
Experimental apparatus for unconfined compression test and direct shear test of cemented soil. (**a**) Rock mechanics testing machine; (**b**) Direct shear apparatus.

A rock mechanics testing machine (Figure 6a) was used for the unconfined compression tests of the cemented soil. The testing specimens were cubes with sizes of 70.7 × 70.7 × 70.7 mm. The direct shear apparatus (Figure 6b) was used to measure the shear strength of the cemented soil samples. Thirty groups of cemented soil cutting ring specimens with sizes of 61.8 × 20 mm (diameter × height) were prepared.

**Figure 7:**
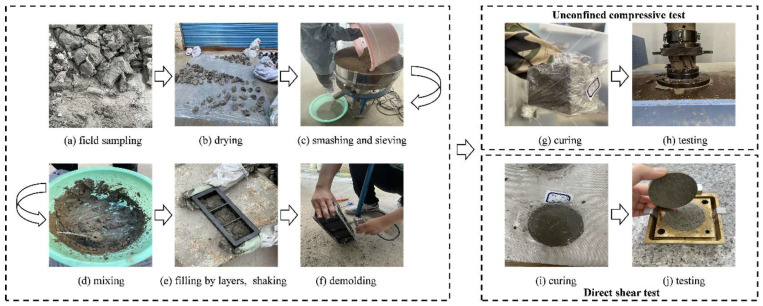
Specimen preparation procedures of unconfined compressive and direct shear tests of cemented soil.

Similarly, specimen quality control was very important for the experimental results. All the small cemented soil specimens for the unconfined compression test and the direct shear test were prepared during the preparation of the large-scale interface shear test specimens for consistency, as shown in Figure 7.

## 3. Results and Discussion

### 3.1. Shear Strength of Cemented Soil–Concrete Interface

**Figure 8:**
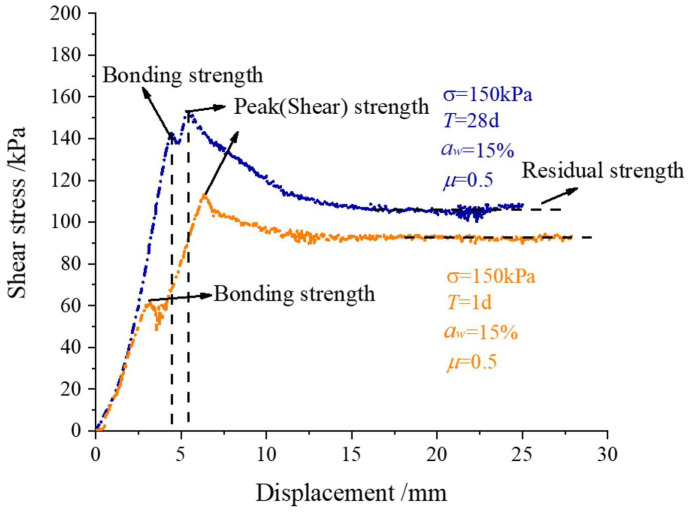
Shear stress–displacement curve of cemented soil–concrete interface with basic reference parameter values from large-scale shear tests.

**Figure 9:**
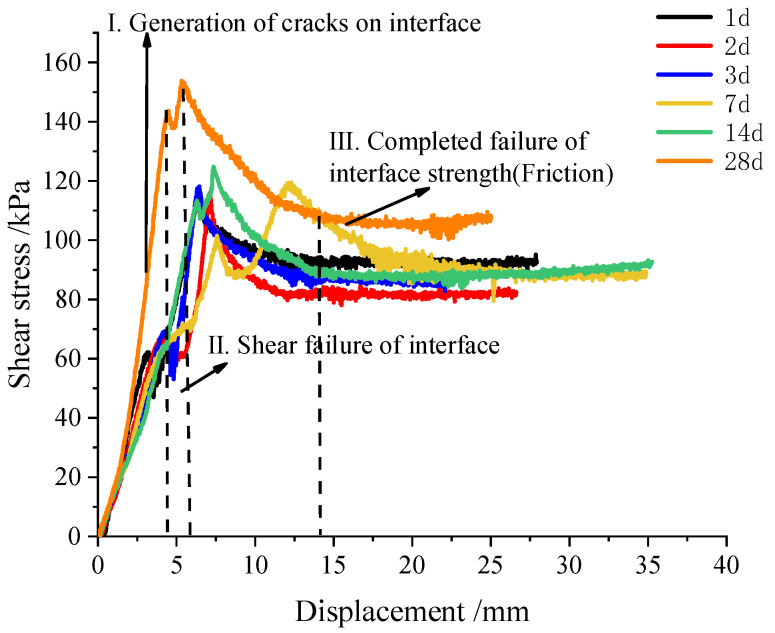
Shear stress–displacement curve of concrete–cemented soil interface from large-scale shear tests.

**Figure 10:**
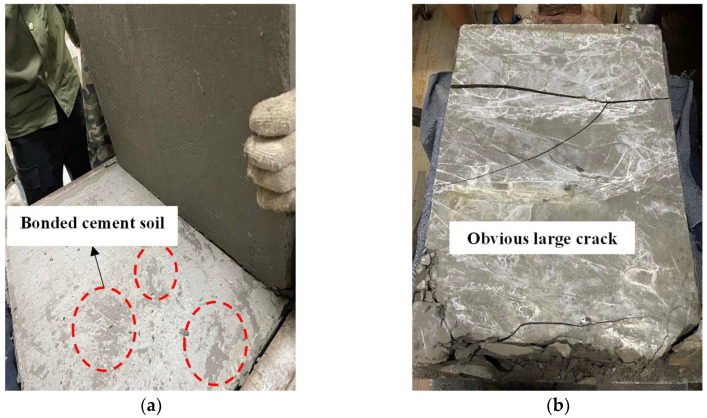
Different occurrences of interfaces after shear stress at difference ages. (**a**) Only broken bonding surface, no obvious internal cracks (age of 1 d). (**b**) Interface totally destroyed, obvious cracks (age of 28 d).

The shear stress–displacement development characteristics of the concrete-cemented soil samples aged 1 d and 28 d are shown in Figure 8. From the shear stress–displacement curve, they both show a typical kind of brittle failure mode. The maximum shear stress could be taken as the shear strength of the cemented soil–concrete interface, as shown in Figure 8, while the shear stress–displacement curves of the interface have a small peak before the maximum value of shear stress. This is presented at the end of the linear stage of the curve. All the shear stress–displacement data of the interface at different ages of 1~28 d are all presented in Figure 9. It can be found that all the curves present a small peak strength. Additionally, with a longer curing age, this small peak of interface shear strength is larger. The occurrence of this shear strength before the peak shear strength is due to the bonding effect of cemented soil and concrete during sample preparation, i.e., cemented soil was cast in place on a precast concrete mold, and not prepared separately. Actually, the preparation of casting-in-place was much more consistent with real engineering construction. Therefore, this small peak in shear strength is regarded as bonding shear strength (Figure 8). It is important to understand the shearing mechanism of a cemented soil–concrete interface when small displacement is encountered, which is also the main difference with previous studies on cemented soil–concrete interface shear strength, where only peak shear strength occurs. It is considered that the small peak of bonding strength presents a certain degree of unloading caused by the generation of small cracks on the interface. This indicates that it is due to the previous destruction of the bonding effect, not the interface itself. Aa shown in Figure 10, under different ages of cemented soil, i.e., different bonding effects, the failure occurrence at the interface after shearing is totally different, as observed in the cracks aged 1 d and 28 d. 

When the displacement continues to increase, the cemented soil and concrete are compacted again, and the shear stress continues to increase. When the shear stress reaches the maximum value, the interface is destroyed (Figure 10b). The shear stress becomes stable when the interface is completely destroyed and the rough surface is smoothed via shearing. This stable value can be defined as residual shear strength (Figure 8). Friction plays a main role in residual strength. 

**Figure 11:**
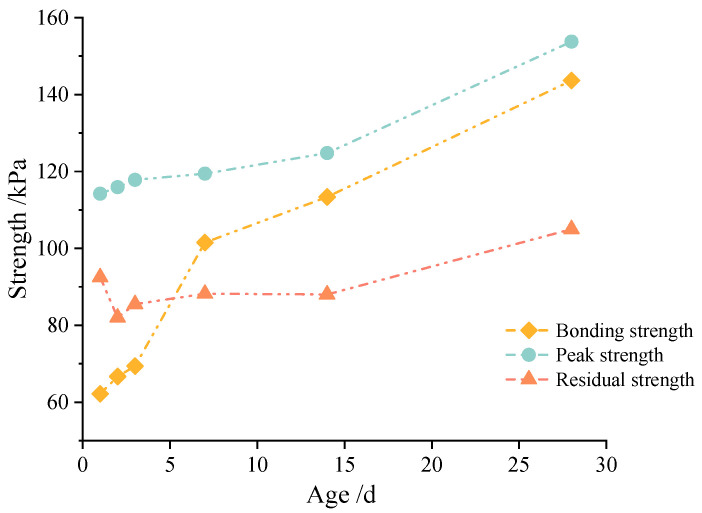
Bonding strength, peak strength and residual strength of the interface at different ages from large-scale shear tests.

Generally, based on our observations and the above analysis of the shear stress–displacement characteristics, the failure process of the cemented soil–concrete interface can be divided into three parts: (1) the generation of small cracks at the interface; (2) the interface is destroyed and shear failure occurs; and (3) the interface is completely cracked, and the residual strength mainly consists of friction. Correspondingly, the bonding shear strength, peak shear strength and residual shear strength of the cemented soil–concrete interface are defined, and all the specific values of the cemented soil–concrete interface at different ages, the cement mixing ratio, the water–cement ratio and normal stress are presented in Table 7. It is found that the shear strength of the interface increases with the age, the cement mixing ratio and normal stress, and decreases with an increase in the water–cement ratio. At the same time, the interface shear strength grows rapidly during at 14 d to 28 d. Additionally, from Figure 11, it can be seen that the peak strength and residual strength have similar trends. They all increase slowly in the early stage before 14 d, but have rapid growth at 14 d to 28 d. On the contrary, the bonding strength increases rapidly from 1 d to 7 d. Additionally, the rate of growth becomes slower from 7 d to 28 d.

### 3.2. Unconfined Compressive Strength and Shear Strength of Cemented Soil

The unconfined compressive strength and shear strength of the cemented soil samples are shown in Table 8.

**Figure 12:**
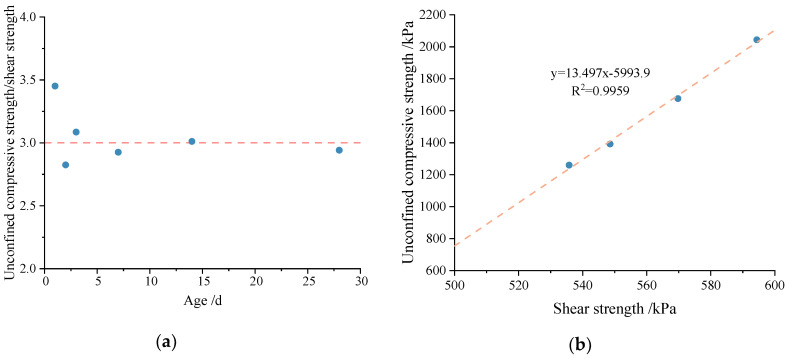
Relationship between unconfined compressive strength and shear strength of cemented soil. (**a**) Ratio of unconfined compressive strength to shear strength of cemented soil at different ages. (**b**) Relationship between unconfined compressive strength and shear strength of cemented soil at the same age of 28 d.

The results of Table 8 indicate that the unconfined compressive strength and shear strength are positively correlated, and both of them increase with age. They also increase with increases in the cement mixing ratio and decrease with increases in the water–cement ratio. Additionally, the shear strength increases with normal stress. From the perspective of age, the ratio of unconfined compressive strength to shear strength is around 3.0, as shown in Figure 12a. To some extent, age is not the key factor that influences the relationship between unconfined compressive strength and shear strength. For different types of cemented soil samples at the same age of 28 d, there is a positive linear correlation between unconfined compressive strength and shear strength, as shown in Figure 12b. For the shear strength index of cemented soil, it is obvious that cohesion is positively correlated with unconfined compressive strength and shear strength. The friction angle increases relative to age and the water–cement ratio, and decreases with the cement mixing ratio.

### 3.3. Failure Mechanism of Cemented Soil–Concrete Interface

To better understand the failure mechanism of the cemented soil–concrete interface, the relationship between the cemented soil–concrete interface shear strength and the unconfined compressive strength of the cemented soil, the cemented soil–concrete interface shear strength and the shear strength of the cemented soil was analyzed, under a same normal stress of 150 kPa, at different ages. The comparison indicates that the cemented soil–concrete interface shear strength at any stage of curing is always smaller than the cemented soil’s own shear strength, as shown in Table 9. This is probably to say that the bonding effect of the cemented soil–concrete interface is weaker than the strength of the cemented soil particles. During the shear process, the interface of cemented soil–concrete must be the weak surface, instead of the cemented soil. So, when the cemented soil–concrete interface that forms in some underground constructions is subjected to shear action, the interface will be destroyed before the cemented soil or concrete. Additionally, the value of the unconfined compressive strength is much greater than that of the shear strength of the interface and cemented soil under the same condition. Additionally, the shear strength and unconfined compressive strength of cemented soil increase more quickly than the interface shear strength with curing duration. 

**Figure 13:**
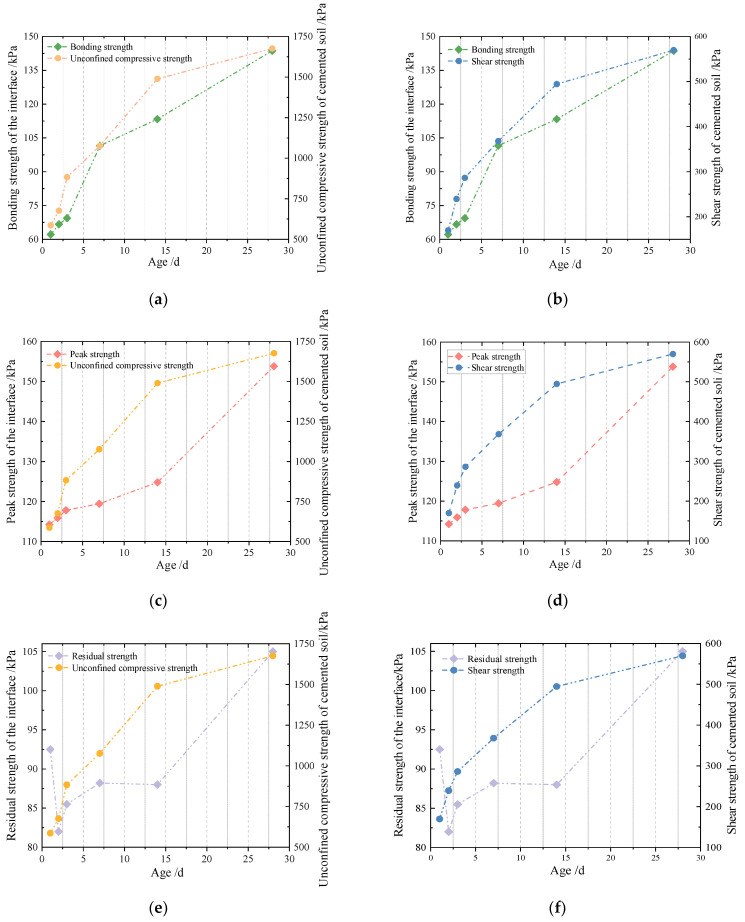
Relationship between bonding strength, peak strength and residual strength of the interface and unconfined compressive strength or shear strength of cemented soil. (**a**) Bonding strength of the interface and unconfined compressive strength of cemented soil. (**b**) Bonding strength of the interface and shear strength of cemented soil. (**c**) Peak strength of the interface and unconfined compressive strength of cemented soil. (**d**) Peak strength of the interface and shear strength of cemented soil. (**e**) Residual strength of the interface and unconfined compressive strength of cemented soil. (**f**) Residual strength of the interface and shear strength of cemented soil.

From Table 9, it can be figured out that the shear strength of the cemented soil–concrete interface is positively related to the unconfined compressive strength and shear strength of the cemented soil. With an increase in unconfined compressive strength and shear strength, the shear strength of the cemented soil–concrete interface will increase correspondingly. As shown in the above analysis of Figure 11, it can be easily seen that the trends of bonding strength and peak and residual strength are different, although all of them are positively related to unconfined compressive strength and shear strength. Upon looking further, the trends of bonding strength and unconfined compressive strength or shear strength are much closer than those of peak and residual strength, as shown in Figure 13. That is, it grows fast in the early age and slows down from 7 d to 28 d. In principle, the increase in the unconfined compressive strength and shear strength of cemented soil is mainly related to the cementation of cement hydration products. In the initial stage of curing, the hydration of cement can proceed quickly and react adequately due to sufficient materials, water, etc. The hydration products form cementation with other components and the strength of the cemented soil grows rapidly. With the continuous development of time, the water and reaction materials decrease. So, hydration slows down, and the strength growth of the cemented soil slows down as well. At the same time, the bonding strength of the interface also represents the degree of cementation. So, the relationship between the bonding strength and the unconfined compressive strength or shear strength of cemented soil is closer. On the other hand, after reaching the bonding strength, the cementation of the cemented soil gradually disappears. So, the peak and residual strength relate more closely to the properties of the interface itself rather than cemented soil material. At this point, the particle arrangement and friction characteristics at the interface may affect the peak (shear) strength and residual strength even more. This allows both to have the same rapid growth from 14 to 28 days. As a result, the curing influence on interface shear strength and unconfined compressive strength or shear strength are not close, although there is positive relationship between peak and residual strength and unconfined compressive strength or shear strength. 

## 4. Conclusions

To figure out the failure mechanism and characteristics of cemented a soil–concrete interface, a series of large-scale contact surface shear tests and corresponding unconfined compressive tests and direct shear tests of cemented soil were carried out. The following important conclusions can be drawn:

1. Three stages of the failure process of the cemented soil–concrete interface can be observed: (1) the generation of cracks at the interface; (2) the interface is destroyed and shear failure occurs; (3) the interface completely fails, and the residual strength mainly consists of friction. The bonding strength, peak (shear) strength and residual shear strength of the cemented soil–concrete interface are correspondingly proposed and analyzed.
2. The shear strength of the interface increases with the age, cement mixing ratio and normal stress, and decreases with an increase in the water–cement ratio. Additionally, the interface shear strength grows rapidly at 14 d to 28 d. Hence, the cemented soil used in some practical projects needs to be cured for a sufficient amount of time, such that the strength of the cemented soil–concrete interface can be improved sufficiently.
3. The results of the unconfined compressive test and direct shear test on the cemented soil indicate that the unconfined compressive strength and shear strength are positively correlated. Additionally, both of them increase with age and the cement mixing ratio and decrease with an increase in the water–cement ratio. There is a positive correlation between interface shear strength and the unconfined compressive strength or shear strength of cemented soil. However, the trends of the interface bonding strength and unconfined compressive strength or shear strength of cemented soil are much closer than those of the interface peak strength and unconfined compressive strength or shear strength, and the interface residual strength and unconfined compressive strength or shear strength. This is considered to be related to the cementation of cement hydration products and probably the particle arrangement of the interface and its friction. Additionally, interface strength is always smaller than the unconfined compressive strength or shear strength of the cemented soil itself.

This research is of great significance to the study of the contribution of materials’ strength to interface evolution at different stages. However, the effects of age, the cement mixing ratio, the water–cement ratio and normal stress on the cementation and shear properties of cemented soil–concrete interfaces have not been analyzed at the microscopic level. That is to say, the essence is not clear. So, further studies on the inherent mechanism for interface strength improvement, and tests of more influencing factors on the cemented soil–concrete interface, are expected.

## Figures and Tables

**Table 1:** Basic properties of soil sample.

Water Content *ω*/%	Density *ρ*/g cm^−3^	Void Ratio *e*	Liquidity Index *I_L_*	Plasticity Index *I_P_*	Liquid Limit *ω_L_*/%	Plastic Limit *ω_P_*/%	Shear Strength *	
							Cohesion *c*/kPa	Friction Angle *φ*/°
49.3	1.73	1.36	1.35	19.0	42.7	23.7	11.90	8.00

Shear strength *: from the direct shear test.

**Table 2:** Properties of the cement.

Dry Density/g·cm^−3^	Fineness/%	Initial Setting Time/min	Final Setting Time/min	Compressive Strength (28 d)/MPa	Flexural Strength (28 d)/MPa
3.1	1.1	130	210	43.5	7.8

**Table 3:** Concrete proportion design.

Water–Cement Ratio	Cement/kg	Ground Sand/kg	Sand/kg	Stone/kg	Water Reducing Agent/kg
0.3	301	129	697	1250	14

**Table 4:** Recommendations for cement mixing ratio and water–cement ratio in specifications.

Specification	Cement Mixing Ratio/%	Water–Cement Ratio
Specification for mixed proportion design of cement soil (JCJ/T 233-2011) [26]	3~25	0.45~2.0
Technical code for composite foundation (GBT50783-2012) [23]	10~20	-
Technical specification for pile foundation of pipe pile embedded in cemented soil (JGJ/T 330-2014) [24]	≥20	0.8~1.5
Technical specification for strength composite piles (DGJ32/TJ 151-2013) [22]	15~25	0.8~1.2
Technical code for excavation engineering (DG/TJ 08-61-2010) [30]	Biaxial cement mixing pile: 13~15 Triaxial cement mixing pile: 20~22	0.5–0.6

**Table 5:** Experimental program of large-scale interface shear tests.

Age/d	Cement Mixing Ratio/%	Water–Cement Ratio	Normal Stress/kPa
1	15	0.5	150
2	15	0.5	150
3	15	0.5	150
7	15	0.5	150
14	15	0.5	150
28	15	0.5	150
28	13	0.5	150
28	18	0.5	150
28	15	0.8	150
28	15	0.5	50
28	15	0.5	250

**Table 6:** Experimental program of unconfined compression test and direct shear test of cemented soil.

Experiments	Age/d	Cement Ratio/%	Water–Cement Ratio	Normal Stress/kPa
Unconfined compression test	1, 2, 3, 7, 14, 28	15	0.5	-
	28	13	0.5	-
	28	18	0.5	-
	28	15	0.8	-
	28	15	1.0	-
Direct shear test	1, 2, 3, 7, 14, 28	15	0.5	50, 150, 250
	28	13	0.5	50, 150, 250
	28	18	0.5	50, 150, 250
	28	15	0.8	50, 150, 250
	28	15	1.0	50, 150, 250

**Table 7:** Shear strength of concrete–cemented soil interface.

Age/d	Cement Mixing Ratio/%	Water Cement Ratio	Normal Stress/kPa	Bonding Strength/kPa	Shear (Peak) Strength/kPa	Residual Strength/kPa
1	15	0.5	150	62.158	114.216	92.5
2	15	0.5	150	66.667	115.908	82.0
3	15	0.5	150	69.382	117.828	85.5
7	15	0.5	150	101.522	119.449	88.2
14	15	0.5	150	113.359	124.793	88.0
28	15	0.5	150	143.665	153.786	105
28	13	0.5	150	75.193	140.292	90.5
28	18	0.5	150	81.703	163.978	118.8
28	15	0.8	150	110.090	135.825	101.0
28	15	0.5	50	23.450	62.296	39.6
28	15	0.5	250	203.693	238.815	198.5

**Table 8:** Unconfined compressive strength and shear strength of cemented soil.

Age/d	Cement Mixing Ratio/%	Water-Cement Ratio	Normal Stress/kPa	Unconfined Compressive Strength/kPa	Shear Strength/kPa	Cohesion *c*/kPa	Friction Angle *φ*/°
1	15	0.5	150	586.18	169.83	65.93	34.68
2	15	0.5	150	676.28	239.43	135.57	34.56
3	15	0.5	150	883.35	286.29	173.70	35.90
7	15	0.5	150	1076.31	367.92	257.19	38.16
14	15	0.5	150	1489.29	494.49	366.04	44.63
28	15	0.5	150	1675.95	569.75	408.03	45.59
28	13	0.5	150	1392.14	548.56	363.63	46.83
28	18	0.5	150	2043.90	594.37	450.47	43.67
28	15	0.8	150	1259.98	535.78	362.80	49.05
28	15	1.0	150	938.08	501.68	341.88	50.04
28	15	0.5	50	1675.95	454.76	408.03	45.59
28	15	0.5	250	1675.95	658.91	408.03	45.59

**Table 9:** Strength of cemented soil–concrete interface, unconfined compressive strength and shear strength of cemented soil at different times (under a same normal stress of 150 kPa).

Age/d	Cemented Soil–Concrete Interface			Cemented Soil	
	Interface Shear (Peak) Strength/kPa	Bonding Strength/kPa	Residual Strength/kPa	Shear Strength/kPa	Unconfined Compressive Strength/kPa
1	114.216	62.158	92.5	169.83	586.18
2	115.908	66.667	82.0	239.43	676.28
3	117.828	69.382	85.5	286.29	883.35
7	119.449	101.522	88.2	367.92	1076.31
14	124.793	113.359	88.0	494.49	1489.29
28	153.786	143.665	105.0	569.75	1675.95

## Data Availability

Not applicable.
